# Analysis on Blast Fungus-Responsive Characters of a Flavonoid Phytoalexin Sakuranetin; Accumulation in Infected Rice Leaves, Antifungal Activity and Detoxification by Fungus

**DOI:** 10.3390/molecules190811404

**Published:** 2014-08-04

**Authors:** Morifumi Hasegawa, Ichiro Mitsuhara, Shigemi Seo, Kazunori Okada, Hisakazu Yamane, Takayoshi Iwai, Yuko Ohashi

**Affiliations:** 1 College of Agriculture, Ibaraki University, 3-21-1 Chuo, Ami, Ibaraki 300-0393, Japan; E-Mail: morifumi@mx.ibaraki.ac.jp; 2 National Institute of Agrobiological Sciences, Tsukuba, Ibaraki 305-8602, Japan; E-Mail: sseo71@affrc.go.jp; 3 Biotechnology Research Center, The University of Tokyo, 1-1-1 Yayoi, Bunkyo-ku, Tokyo 113-8657, Japan; E-Mail: ukokada@mail.ecc.u-tokyo.ac.jp; 4 Department of Biosciences, Teikyo University, 1-1 Toyosatodai, Utsunomiya, Tochigi 320-8551, Japan; E-Mail: hyamane@nasu.bio.teikyo-u.ac.jp; 5 School of Food, Agricultural and Environmental Sciences, Miyagi University, 2-2-1 Hatadate, Taihaku, Sendai, Miyagi 982-0215, Japan; E-Mail: iwaitk@myu.ac.jp

**Keywords:** antifungal activity, blast fungus, detoxification, flavonoid, hypersensitive reaction (HR), momilactone A, phytoalexin (PA), resistance, rice, sakuranetin

## Abstract

To understand the role of the rice flavonoid phytoalexin (PA) sakuranetin for blast resistance, the fungus-responsive characteristics were studied. Young rice leaves in a resistant line exhibited hypersensitive reaction (HR) within 3 days post inoculation (dpi) of a spore suspension, and an increase in sakuranetin was detected at 3 dpi, increasing to 4-fold at 4 dpi. In the susceptible line, increased sakuranetin was detected at 4 dpi, but not at 3 dpi, by which a large fungus mass has accumulated without HR. Induced expression of a PA biosynthesis gene *OsNOMT* for naringenin 7*-O*-methyltransferase was found before accumulation of sakuranetin in both cultivars. The antifungal activity of sakuranetin was considerably higher than that of the major rice diterpenoid PA momilactone A *in vitro* and *in vivo* under similar experimental conditions. The decrease and detoxification of sakuranetin were detected in both solid and liquid mycelium cultures, and they took place slower than those of momilactone A. Estimated local concentration of sakuranetin at HR lesions was thought to be effective for fungus restriction, while that at enlarged lesions in susceptible rice was insufficient. These results indicate possible involvement of sakuranetin in blast resistance and its specific relation to blast fungus.

## 1. Introduction

Phytoalexins (PAs) are induced by stresses, including microbial infection, in many plant-microbe interactions, and are defined as low molecular weight antifungal compounds biosynthesized *de novo* by host plants [1,2,3]. PAs have diverse structures, including flavonoid, isoflavonoid, diterpenoid, sesquiterpenoid, polyacetylenes, indoles and stilbenoid, and the importance as general defense compounds was demonstrated in dicot plants such as tobacco, tomato and alfalfa by ectopic expression of a PA biosynthesis gene [4]. At the same time, metabolism and detoxification of PAs by the pathogenic fungus, which are thought to be a way to overcome the defense of plants, were reported in dicot plants [5]. In monocot plants, characteristic natures and roles of diterpenoid PAs for defense were indicated [6], however many subjects remain to be studied. In blast fungus-infected rice leaves, accumulation of sixteen PAs, including fifteen diterpenes such as momilactone A and one flavonoid PA, sakuranetin, was reported [7,8]. A previous study on the major rice diterpenoid PA momilactone A indicated the importance for blast resistance via experiments on its time-dependent accumulation profiles in resistant and susceptible rice lines, antifungal activity of the PA *in vitro* and *in vivo*, and detoxification of the PA by the fungus in mycelium cultures [9].

Manuscripts in the 1990s on the rice flavonoid PA sakuranetin described that blast fungus infection induced higher levels of sakuranetin in resistant rice lines than in susceptible rice lines [10,11]. However, the experimental systems used for the sakuranetin quantification contained a wound process, which itself can induce PA increases. In the former study [10], a spore suspension was applied on the finger-rubbed leaf surface of detached leaves. After an appropriate time, droplets on the leaves were collected and combined with excised leaf discs which contained fungus-induced spots, and subjected to sakuranetin quantification. In the later paper [11], a spore suspension was applied after crushing the leaf surfaces with a pair of headless pliers and the leaf was used for the quantification. These procedures were accompanied by severe wounding. Actually, treatment with the defense signal compound jasmonic acid, whose increase is generally induced after wounding, increased sakuranetin accumulation [12]. To reveal the net increase in sakuranetin caused by blast fungus infection, experiments should be conducted under natural infection conditions eliminating the wound effect. At the same time, experiments to reveal its antifungal activity and detoxification by blast fungus are necessary to provide information about the defensive characteristics of the PA against fungi.

Thus, we here studied these using resistant and susceptible rice lines and a corresponding blast fungus race. The results indicate that: (1) sakuranetin has stronger antifungal activity to blast fungus than the diterpenoid PA momilactone A *in vitro* and *in vivo*; (2) the concentration of sakuranetin accumulated in infected regions in the resistant rice line was estimated to be effective enough to restrict the fungus, while that in susceptible rice was too low for it; (3) blast fungus converted sakuranetin to less toxic compounds as did momilactone A, while the rate of conversion was slower than that of momilactone A. These results indicate the fungus-responsive characteristics of sakuranetin, and the defensive role of the PA in the fight of host rice plants *vs.* invaded blast fungus.

## 2. Results and Discussion

### 2.1. The Local Concentration of Sakuranetin is Estimated to be Superior in Infected Regions in a Resistant Rice Line than in a Susceptible Line

Sakuranetin is a rice flavonoid PA with a flavanone structure, and its increase by blast fungus infection has been reported by Kodama *et al.* [10] and Dillon *et al.* [11]. They used pre-wounded rice leaves for fungal inoculation. To evaluate the net increase in the PA levels caused by fungus infection, experiments should be done under natural infection conditions without any wound effect. Then, we spray-inoculated intact rice seedlings grown in soil with a spore suspension of blast fungus race 003, and the 4th leaves were subjected to sakuranetin quantification. In this experimental system, typical susceptible and resistant responses of host rice plants to the fungal type were observed [9,13]; blast fungus grows vigorously in the susceptible rice line Nipponbare (N), and susceptible type enlarged light brown lesions (ELs) become visible at 4 days post inoculation (dpi). On the other hand, in resistant rice line IL7, which was generated by introduction of a rice resistance gene *Pii* to the fungus into N and back-crossings [14], fungal growth is severely restricted during the early infection period [9,13] accompanying hypersensitive reaction (HR), which is a typical resistance response of host plants to restrict invasive pathogens to infected regions. HR generally accompanies formation of HR lesions (HRLs), which are very small size and dark brown and become visible at 2 dpi, increasing the number at 3 dpi [9].

**Figure 1:**
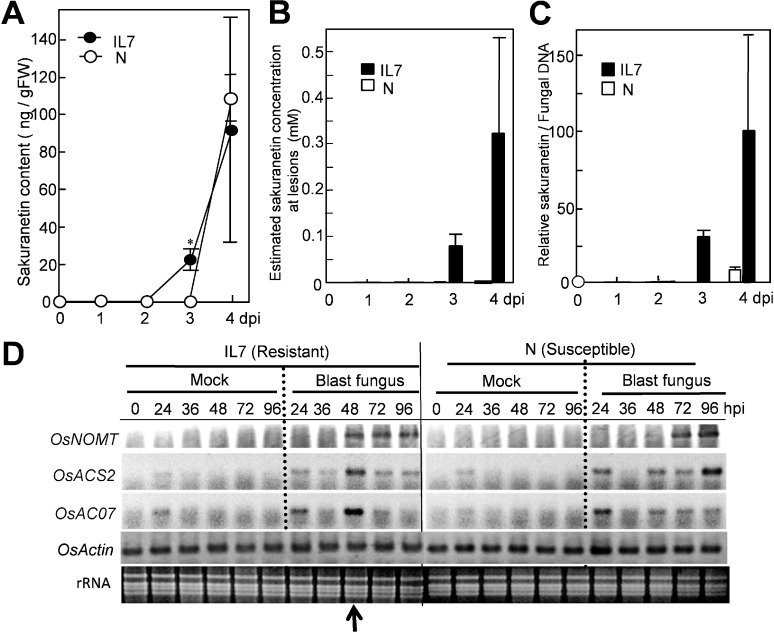
Increase in sakuranetin and transcript of *NOMT* in resistant IL7 and susceptible N rice lines after inoculation with blast fungus. (**A**) Sakuranetin content in leaves; (**B**) Estimated sakuranetin concentration at lesions; (**C**) Relative content of sakuranetin/fungal DNA; (**D**) RT-PCR analysis on a sakuranetin biosynthesis gene *OsNOMT* for naringenin 7*-O*-methyltransferase and HR-inducible control genes *OsACS2* and *OsACO7* in rice leaves after mock- or blast-inoculation according to Iwai *et al.* [13]. Arrow indicates the time of HR lesion visualization. Asterisks indicate a significant difference between inoculated N and IL7 leaves at 3 dpi (Student’s paired *t* test: *p* < 0.01). gFW: g fresh leaf weight. dpi: Day post inoculation. hpi: Hour post inoculation. Data are means ± standard deviation (SD) from independent three samples for Figure 1A–C.

After spray-inoculation with a spore suspension, only a small amount of sakuranetin at a background level was found at 0–2 dpi in both susceptible N and resistant IL7 rice leaves (Figure 1A). An increase in sakuranetin was first found in IL7 at 3 dpi, and was estimated to be 23 ng per g fresh leaf (closed circles), and the level increased to 92 ng at 4 dpi. In the N leaves, the increase was not detected at 3 dpi, and 108 ng was found at 4 dpi (open circles). The majority of PAs have been reported to accumulate locally at fungus-induced lesions [15,16], and the area occupied by HRLs at 3 dpi was roughly estimated to about 0.1% of the whole IL7 leaf area; around 50 HRLs with about 0.01 mm^2^ each were formed in the leaf of 500 mm^2^ [9,17] indicating that about 1,000-fold higher sakuranetin than the mean level is localized at HRLs. As the mean sakuranetin content in IL7 at 3 dpi was 23 ng per g of fresh leaf, the local concentration at HRLs was 23 μg per g leaf corresponding to near 0.1 mM (Figure 1). At 4 dpi, the mean PA content was 92 ng per g of IL7 leaf, so the local concentration of HRLs was estimated to be about 0.3 mM. In susceptible N leaves, the area occupied by susceptible type enlarged lesions (ELs) was about 20% of the whole leaf at 4 dpi with accumulation of a large fungus mass [9,17]. Mean sakuranetin content at 4 dpi was 108 ng per g of N leaf, and accordingly the local concentration at ELs was estimated to 540 ng per g leaf corresponding to 2 μM (Figure 1B, N: open circles). These results suggest that the net sakuranetin concentration at fungus-induced lesions was estimated to be about 160-fold higher in IL7 than N at 4 dpi. The sakuranetin contents quantified here were converted into relative values based on fungal DNA contents in infected leaves [9], demonstrating that those were much higher in IL7 than in N (Figure 1C). 

The sakuranetin contents in the resistant rice line detected here were considerably lower than the levels mentioned in previous reports; 100 μg per g leaf at 2 dpi by Kodama *et al.* [10] and 15.8 μg per g leaf at 3 dpi by Dillon *et al.* [11]. As indicated by Tamogami *et al.* [12], treatment with the wound hormone jasmonic acid elicited 40-fold more sakuranetin in rice leaves in comparison with water-treated control. Then, one of the possible reasons for the different results could come from the wound-inducible nature of sakuranetin. Actually, in our experimental system, sakuranetin was detected in a very small amount in healthy rice leaves, and increased about 30-fold by cutting leaves at 48 h. We spray-inoculated a fungal spore suspension and inoculated rice seedlings were carefully grown without wounding before sakuranetin quantification. However, in the previous papers, the inoculation methods involved severe wounding procedures. Another reason for the different results could come from the different experimental system used, such as rice lines, fungus race, incubation conditions and quantification methods. 

Next, induced expression of a sakuranetin biosynthesis gene upon HR was studied by RT-PCR according to Iwai *et al.* [13]. Expression of the *OsNOMT* gene for naringenin 7*-O*-methyltransferase which is a key enzyme in the biosynthesis of sakuranetin (Shimizu *et al.* [18]) was analyzed with that of HR inducible positive control genes, *OsACS2* and *OsACO7* for ethylene biosynthesis [13]. The transcripts for *OsACS2* and *OsACO7* were transiently accumulated in blast inoculated IL7 at 48 h post inoculation (hpi) at which HRLs became to be visible but not in inoculated N (Figure 1D). Transcript of *OsNOMT* was found in IL7 but not in N at 48 hpi. Expression time of *OsNOMT* in IL7 was 48–96 hpi in IL7, while it was at 72–96 hpi in N, suggesting induction of *OsNOMT* preceded sakuranetin accumulation in both lines. 

### 2.2. Inhibition of Fungal Growth by Sakuranetin in Vitro and in Planta

The inhibitory activity of sakuranetin to mycelium growth was studied *in vitro* using a solid medium. Four blast mycelium plugs (about 10 mm^2^ each) were inoculated on potato dextrose agar (PDA) containing sakuranetin at various concentrations, and fungal growth was analyzed measuring the diameter of the mycelium colony after appropriate incubation. 

**Figure 2:**
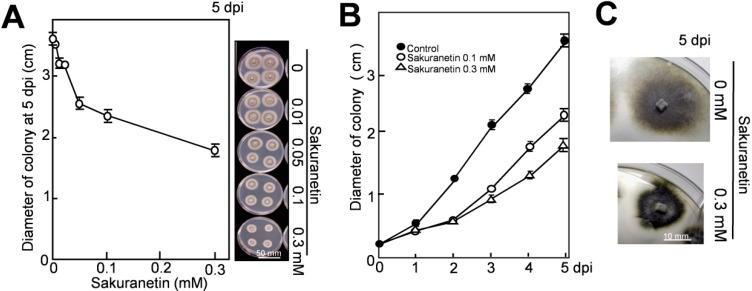
Inhibition of blast mycelium growth by sakuranetin on solid medium. (**A**) Size of blast mycelium colony on PDA containing sakuranetin at 5 dpi; (**B**) Time course analysis on the spread of blast mycelium colony on PDA containing sakuranetin at 0.1 or 0.3 Mm; (**C**) Phenotype of the mycelium colony with or without 0.3 mM sakuranetin at 5 dpi. Data are means ± SD from four independent samples. Scale bar in Figure 2A indicates 50 mm, and that in Figure 2C 10 mm.

As shown in Figure 2A, sakuranetin inhibited fungal mycelium growth in a concentration-dependent manner on PDA. On the right side, the phenotypes of mycelium colonies at 5 dpi are shown. A time-course experiment indicates that growth of mycelium colony in diameter at 2 dpi was inhibited about 50% by sakuranetin at both 0.1 and 0.3 mM (Figure 2B). The inhibition rate by 0.3 mM sakuranetin became slightly higher than by 0.1 mM thereafter, and 51 and 36% of the growth was inhibited by 0.3 and 0.1 mM at 5 dpi, respectively. The level of antifungal activity on PDA was similar to that on potato sucrose agar (PSA), which contains sucrose instead of glucose as the naturally occurring sugar source in plants. The phenotype of the mycelium colony was altered in the presence of sakuranetin. The colony looked like more condensed and risen on both PDA and PSA containing 0.3 mM sakuranetin, and an example on PSA at 5 dpi is shown in Figure 2C.

Referring to the report by Hasegawa *et al.* on the diterpenoid PA momilactone A [9], the antifungal activity of sakuranetin on blast fungus was higher than that of momilactone A under similar experimental conditions. For example, momilactone A inhibited 12% and 17% of the mycelium growth on PDA at 0.1 and 0.3 mM at 4 dpi respectively [9], while sakuranetin inhibited 40% and 55% of it at 0.1 and 0.3 mM, respectively (Figure 2B). 

**Figure 3:**
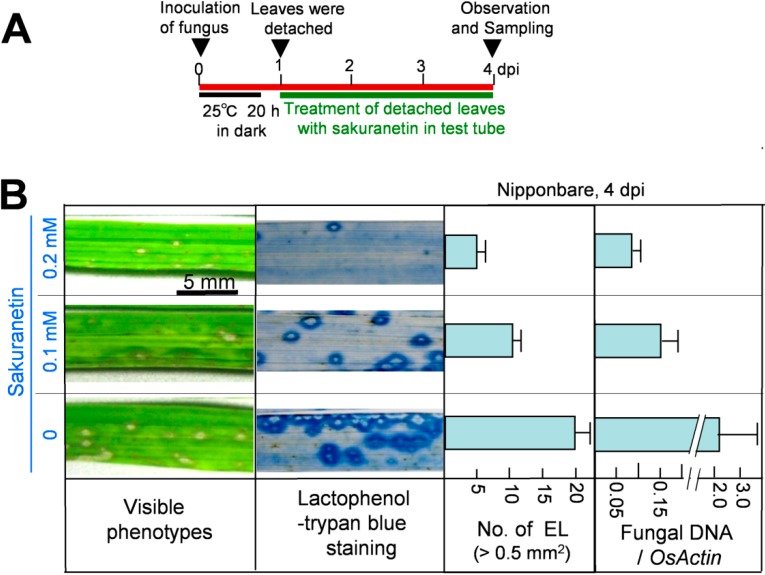
Inhibition of blast fungus growth by sakuranetin in rice leaves at 4 dpi. (**A**) Experimental protocol; (**B**) Leftmost column: visible lesion phenotype on blast-inoculated Nipponbare leaves treated with sakuranetin; second column: phenotype after staining mycelium in inoculated leaves with lactophenol-trypan blue; third column: number of ELs larger than 0.5 mm^2^ in a leaf. Rightmost column: fungal DNA content/transcript of *OsActin*. Data are means ± SE from ten independent samples for ELs and three samples for fungal DNA, respectively.

Next, the antifungal activity of sakuranetin was analyzed *in planta*. After spray inoculation with a blast spore suspension, ten 4th leaves from intact susceptible N seedlings were detached at 1 dpi, and the leaf bases were put into a glass tube containing 10 mL of chemical solution, and incubated according to the illustrated method in Figure 3A and in Seo *et al.* [17]. The visible phenotype of inoculated 4th leaves at 4 dpi is shown in the leftmost column of Figure 3B. Compared with the visible phenotype, spread of fungus was more clearly observed after lactophenol-trypan blue staining of mycelium (second column). Sakuranetin treatment at 0.1 and 0.2 mM reduced the number of susceptible type ELs, whose area was more than 0.5 mm^2^, to 50% and 25% respectively (third column) and fungal DNA content to 7% and 5% respectively (most right column) at 4 dpi. When the antifungal activity of sakuranetin *in planta* was compared with that of momilactone A described by Hasegawa *et al.* [9], sakuranetin exhibited higher antifungal activity than momilactone A. For example, the number of ELs in blast-inoculated N leaves was decreased to the 25% of control by 0.2 mM sakuranetin (Figure 3B), and that was decreased to the 43% by 0.2 mM momilactone A [9]. Fungal DNA content in infected leaves was decreased to the 4% by 0.2 mM sakuranetin (Figure 3B), and that was decreased to the 39% by 0.2 mM momilactone A [9]. These results indicate sakuranetin has a considerable antifungal activity *in vitro* and *in vivo*, which is higher than that of momilactone A. The calculated concentration of sakuranetin at HRLs in IL7 seems to be effective to restrict fungal growth, while that at ELs was insufficient in susceptible N (Figure 1B), indicating a possible contribution of sakuranetin for fungal resistance upon HR in IL7. 

### 2.3. Decrease of Sakuranetin in Blast Mycelium Cultures

Detoxification of PA would be a way for the fungus to survive in the invaded host plant. Possible detoxification of sakuranetin by the fungus was examined using both solid and liquid mycelium cultures. Discs 20 mm in diameter of 15-day-old blast mycelium layer on PDA were prepared, and put upside down on new PDA containing 0.3 mM sakuranetin according to the method described by Hasegawa *et al.* [9]. After incubation for the indicated time periods, the agar discs beneath the mycelium discs were prepared and subjected to sakuranetin quantification after treatment with 70% methanol for sakuranetin extraction. 

**Figure 4:**
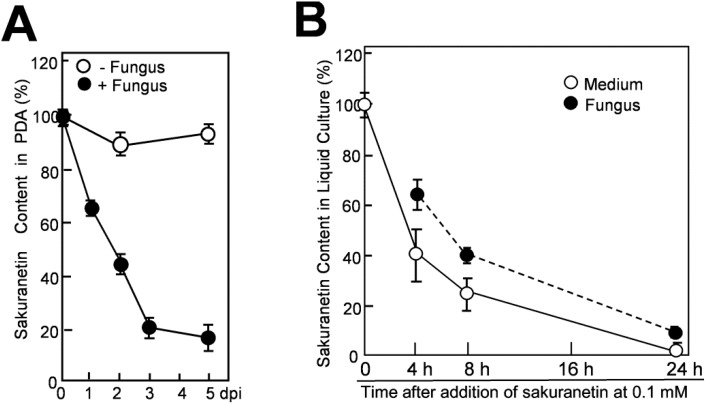
Decrease of sakuranetin in the blast mycelium culture. (**A**) Decrease of sakuranetin content in solid medium PDA after being overlaid by blast mycelium layer (+Fungus) or mock-layer (−Fungus). At time 0, 0.3 mM sakuranetin was present in the agar medium (100%), and sakuranetin in PDA was quantified thereafter; (**B**) Decrease in sakuranetin in liquid mycelium culture. At time 0, 0.1 mM sakuranetin was present in the PD liquid mycelium culture medium (100%). After incubation for the indicated time period, sakuranetin in the medium fraction and fungus mass fraction was quantified separately. Data are means ± SD from three independent samples.

Sakuranetin in the agar medium decreased with time after being overlaid by the mycelium layer. Recovered sakuranetin levels at 1, 2, 3 and 5 dpi decreased to 65%, 45%, 20% and 17% of the initial level, respectively (Figure 4A, closed circles), while no clear decrease was seen after mock-inoculation (open circles). Compared with the data of momilactone A under similar experimental conditions, the rate of decrease of sakuranetin was considerably slower than that of momilactone A; recovered momilactone levels at 1, 2 and 3 dpi were 53%, 3% and 0% of the initial level, respectively [9].

Next, decrease and possible conversion of sakuranetin were analyzed in liquid mycelium culture. The mycelium culture of blast fungus was prepared as described by Hasegawa *et al.* [9]. After addition of sakuranetin at 0.1 mM, 1 mL of culture containing 2 mg equivalent of fungal protein was collected at appropriate time intervals, and fractionated into the supernatant and the precipitated hyphae, designated as medium fraction and fungal mass fraction, respectively. Sakuranetin was then extracted from each fraction with 70% methanol and subjected to quantification. After addition of sakuranetin, about the 60% was transferred to the fungal mass fraction and about the 40% was retained in the medium fraction at 4 h (Figure 4B). The sakuranetin content gradually decreased thereafter in both fractions, and the contents in fungal mass and the medium became 25% and 40% of the initial level, respectively, at 8 h, retaining 65% as total content. At 24 h, it was decreased to 10% in fungal mass and 2% in the medium (Figure 4B).

The decrease in momilactone A using a similar mycelium culture has been reported [9]. However, the rate of decrease was higher than that of sakuranetin. At 4 h after PA application, 35% of the initially added momilactone A remained [9], while the 100% of the sakuranetin remained (Figure 4B). At 8 h, 20% of the momilactone A remained [9], while 65% of sakuranetin was still detected in the culture. These results indicate that sakuranetin is metabolized more slowly by the fungus than momilactone A in both solid and liquid mycelium media. 

### 2.4. Detoxification of Sakuranetin in the Culture of Blast Mycelium

**Figure 5:**
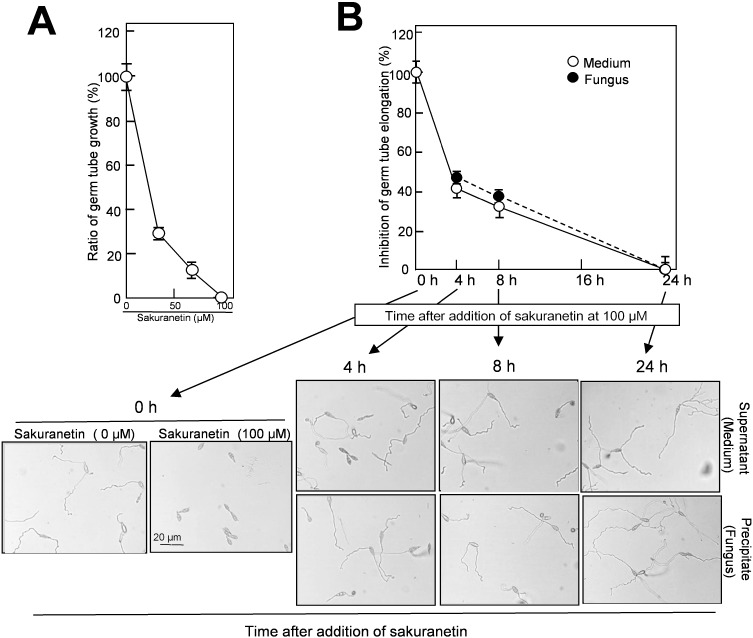
Decrease in antifungal activity in the liquid blast mycelium culture after addition of sakuranetin. (**A**) Standard experiment on germ tube growth inhibition by sakuranetin. Length of germ tubes grown from blast spore was determined at 24 h after incubation with sakuranetin; (**B**) The same lots of samples used in Figure 4B were employed for the antifungal assay. Data are means ± SD from three independent samples each of which contains 10–20 spores for Figure 5A, and from 10 to 20 independent samples each of which contains 5–20 spores for Figure 5B, respectively. The precise method was according to Hasegawa *et al.* [9].

Next, we evaluated whether the decrease in sakuranetin from the liquid mycelium culture accompanies the decrease in antifungal activity or not. As the first experiment, a standard antifungal activity test was conducted using authentic sakuranetin according to the method by Hasegawa *et al.* [9]. In the presence of sakuranetin at 34, 67 and 100 μM, germ tube growth from blast spore was inhibited by 72%, 89% and 100% at 24 h, respectively (Figure 5A). In this system, the sakuranetin concentration required for 50% inhibition of germ tube elongation was estimated to be about 20 μM, which is similar to the concentration reported by Kodama *et al.* [10]. On the other hand, in the presence of momilactone A at 100, 200 and 300 μM, germ tube growth was inhibited 45%, 78% and 96%, respectively [9]. These results indicate that sakuranetin more effectively inhibits fungal growth than momilactone A in liquid culture. 

The same lots of samples which were used for the fungus-induced sakuranetin decrease experiment in liquid culture (Figure 4B), were employed for the antifungal activity analysis. Each sample from the medium and fungal mass fractions was treated with 70% methanol for extraction, and subjected to the assay after concentration. The antifungal activity in both fractions decreased with time, and almost all was lost at 24 h (Figure 5B). A representative photograph used for length determination of elongated germ tube from blast spores is shown at the bottom of Figure 5B. When the antifungal activity found in the medium fraction at time 0 was designated as 100%, the antifungal activity in the medium was decreased to 40% at 4 h, 32% at 8 h and was nearly zero at 24 h, and the antifungal activity in fungal mass fraction decreased to 46% at 4 h, 38% at 8 h and zero at 24 h (Figure 5B). The time-course profile of the loss of antifungal activity was similar to that of the decrease in sakuranetin content shown in Figure 4B, indicating the sakuranetin content in the culture fractions reflects the level of antifungal activity determined by inhibition of germ tube elongation. Thus, the conversion of sakuranetin would accompany its detoxification by blast fungus. The detoxification of sakuranetin may be the second example on fungus-induced conversion of PA from the family Graminaceae. As the first example, Hasegawa *et al.* [9] reported detoxification of momilactone A by blast fungus *in vitro* [9], and Imai *et al.* [19] reported a degradation intermediate of momilactone A. In the mycelium culture after addition of sakuranetin, a small amount of naringenin, which is the precursor of sakuranetin biosynthesis in rice plant, was detected. Quantification and evaluation of naringenin as the metabolite of sakuranetin should be studied in future. Including this subject, isolation and identification of possible metabolites of sakuranetin are in progress. 

### 2.5. Possible Mechanism of Sakuranetin to Restrict Fungus

The results above indicate that superior accumulation of sakuranetin at HRLs in resistant rice contributes to blast resistance. The precise mechanism of how the flavonoid PA sakuranetin inhibits fungal growth is not obvious now. Mizutani *et al.* [20] suggested that a widely used fungicide metominostrobin (SSF-126) is similar in mode of action to CN, which inhibits CN-sensitive respiration in the mitochondrial respiration chain of blast fungus. Although blast fungus then induced CN-resistant respiration to survive, the CN-resistant fungal respiration was inhibited in the presence of synthetic flavone inducing death of the fungus. Seo *et al.* [17] demonstrated that an exogenously supplied synthetic flavone induced blast resistance as well as CN, and further that treatment of CN together with flavone enhanced the resistance *in vitro* and *in vivo*. If the flavonoid PA sakuranetin could inhibit CN-resistant fungal respiration as the flavone did, the PA and CN may co-operationally contribute to the resistance by inhibition of both respiration mechanisms in the fungus. A study to reveal this possibility is in progress. 

Shimizu *et al.* [18] identified the gene for naringenin 7-*O*-methyltransferase (NOMT), which confers the last step of sakuranetin biosynthesis from naringenin. To elucidate the importance of sakuranetin for the resistance, experiments on *NOMT* gene for gain of function in susceptible rice and loss of function in resistant rice would be valuable.

## 3. Experimental Section

### 3.1. Plant Materials and Blast Fungus Inoculation

As susceptible- and resistant-type rice (*Oryza sativa*) lines, Nipponbare (N) and IL7 [14] were used respectively. IL7 is a near isogenic line of N containing the *R* gene *Pii* against blast fungus (*M. oryzae*) race 003 (isolate, Kyu89-241) [21]. Rice plants were grown in cultivation soil (Bonsol No.1, Sumitomo Chem. Ltd., Chuo-ku, Tokyo, Japan) in a growth chamber for 16 h at 450 μmol m^−2^ s^−1^ at 28 °C and 8 h of dark at 25 °C. The 2 week-old seedlings were spray-inoculated with a spore suspension of blast fungus (1 × 10^5^ conidia mL^−1^) containing 0.05% Tween 20, and incubated under dark at high humidity for 20 h at 25 °C for effective infection. The seedlings were moved and incubated in a chamber at 25 °C under 16 h light and 8 h dark cycle. After indicated time periods of incubation, the 4th leaves were used for sakuranetin quantification, fungal DNA quantification and analysis of the number and size of lesions developed. Infected leaves were cut into 20 mm lengths, and subjected to lactophenol-trypan blue staining of mycelium according to Iwai *et al.* [13] after treatment with ethanol.

### 3.2. Quantification of Sakuranetin

For quantification of sakuranetin in blast fungus-inoculated leaves, 0.15 g portions of each of the 4th leaves was dipped in 6 mL of 70% methanol, and heated in a screw cap glass vial for 5 min. At this step, [methyl-^2^H_3_]sakuranetin (40 ng g^−1^ fresh leaf), which was synthesized from naringenin and deuterio-diazomethane according to the method reported by Aida *et al.* [22], was added to the extract to estimate the recovery rate of sakuranetin. Deuteriodiazomethane was prepared by using sodium deuteroxide according to the previously reported method [23,24]. The extract was transferred to a new tube, and the residue was re-extracted twice with 3 mL of 70% methanol. Extracts from three extractions were combined and concentrated to dryness. The residue was dissolved in 0.5 mL of methanol. The solution was centrifuged at 10,000 rpm for 10 min. The supernatant was subjected to the LC/MS/MS to quantify sakuranetin and [methyl-^2^H_3_]sakuranetin by the method described by Shimizu *et al.* [18]. Sakuranetin and [methyl-^2^H_3_]sakuranetin levels were determined with combinations of the precursor and product ions of *m/z* 287/167 for sakuranetin and *m/z* 290/170 for [methyl-^2^H_3_]sakuranetin in the selected reaction monitoring mode.

For quantification of sakuranetin in agar medium culture, each fraction containing sakuranetin was treated with 9 volume of methanol. Sakuranetin was extracted by shaking for 120 min, and 10 μL of the supernatant after centrifugation for 10 min at 10,000 ×*g* was subjected to an HPLC column (Inertsil ODS-3V, 4.6 × 250 mm, GL Science, Shinjuku-ku, Tokyo, Japan) equilibrated with 75% methanol. Sakuranetin was detected by the UV detector at 285 nm after eluting at 1.0 mL/min. For quantification of sakuranetin in liquid mycelium culture, 0.4 mL of methanol was added to 0.1 mL of the solution, and quantified by using LC/MS/MS as described by Inoue *et al.* [8].

### 3.3. Determination of Antifungal Activity of Sakuranetin in Vitro

Blast fungus was cultured on a Petri dish containing PDA at 25 °C in the dark. Fifteen days after incubation, four plugs (10 mm^2^ in area) were prepared from extended front area of mycelium, and inoculated onto PDA containing sakuranetin in a Petri dish 9 cm in diameter. The diameter of mycelium colony was measured after incubation at 25 °C in the dark. 

In liquid culture system, antifungal activity was determined by the inhibition of germ tube elongation from blast spores. Freshly prepared blast spores (4 × 10^3^) were suspended in 100 μL of PD medium containing authentic chemical for standard experiment or the extract from the culture fraction to be tested, and the mixture was put on the center hole (10 mm in diameter) of a glass-bottom dish (35 mm in diameter). After incubation for indicated time period at 25 °C in the dark, photographs of germ tube elongation from blast spores were taken, and they were analyzed by measuring the length of germ tube. The liquid mycelium culture was used according to Hasegawa *et al.* [9]. One mL of the culture containing 2 mg equivalent of fungal protein was prepared at 4, 8 and 24 h, and levels of sakuranetin and antifungal activity in the precipitate and the medium fractions were separately quantified. 

### 3.4. Determination of Antifungal Activity of Sakuranetin in Rice Leaves

Ten fungus-inoculated 4th leaves from 2 week-old seedlings were cut at the base of whole plants at 1 dpi, put into an open 70 mL glass test tube containing 10 mL of chemical solution, and incubated at 25 °C under 16 h light/8 h dark condition. Stock solution of 100 mM sakuranetin in methanol was diluted with water to suitable concentrations, and the same amount of methanol was used for the control. The infected leaves were cut into pieces (2 cm long) before subjecting to lactophenol-trypan blue staining according to Iwai *et al.* [13]. The number of susceptible type enlarged lesion (EL) larger than 0.5 mm^2^ was determined using close-up photographs after staining leaves with lactophenol-trypan blue.

### 3.5. Determination of Blast Fungus DNA in Rice Leaves

Quantification of *M. oryzae* DNA in inoculated leaves was conducted by real-time PCR [25] after extraction with ISO PLANT (Nippon Gene, Chiyoda-ku, Tokyo, Japan). Two specific primer pairs, which were designed based on the 3' non-coding region of a *tubulin* gene in *M. oryzae* (forward, 5'-GGGATGATGGTGGTGGAGGAC-3'; and reverse, 5'-GCCAGGTGCTTAGGACGAAAC-3'). These data were normalized with the DNA amount of a rice *actin* gene (AK060893), which was quantified using following primers (forward, 5'-GAGTATGATGAGTCGGGTCCAG-3'; and reverse, 5'-ACACCAACAATCCCAAACAGAG-3'). 

### 3.6. Analysis of OsNOMT Expression in Mock- and Blast-Inoculated Rice Leaves by RT-PCR

One-step RT-PCR was conducted using the primer sets for the probe of *OsActin* (forward, 5'-GAGAAGAGCTATGAGCTGCCTGATGG-3'; and reverse, 5'-AGGGCAGTGATCTCCTTGCTCAT-3'), and for the probe of *OsNOMT* (forward, 5'-CGGGAGCAGCAGCGGCGAA-3'; and reverse; 5'-GGCGAGCGGTGATCATCCGCA-3'). The precise method and the primer sets for HR-inducible *OsACS2* and *OsACO7* genes were according to Iwai *et al.* [13].

## 4. Conclusions

Blast fungus-responsive characters of the rice flavonoid PA sakuranetin were studied. Increase in sakuranetin was found earlier in resistant IL7 than a susceptible N line after spray-inoculation with a suspension of blast spores. Although the mean sakuranetin concentrations detected in whole IL7 leaves were not so much, the local concentration at HRLs at 4 dpi was estimated to about 0.3 mM, which is an effective concentration to restrict blast fungus. Accumulation of sakuranetin in susceptible rice leaves was not found at 3 dpi. At 4 dpi, the local concentration at the susceptible type ELs was estimated to be about 2 μM, which is insufficient to inhibit fungal growth. The antifungal activity of sakuranetin was determined and compared with that of a major rice diterpenoid PA, momilactone A. Results indicate that the antifungal activity of sakuranetin *in vitro* and also *in vivo* was considerably higher than that of momilactone A under similar experimental conditions. Detoxification of sakuranetin by the fungus in mycelium cultures was found, however it took place more slowly than that of momilactone A. These results reveal the characteristic nature of the flavonoid PA sakuranetin against blast fungus in rice. The level of antifungal activity and the mode of detoxification by blast fungus were different from those of momilactone A, indicating the flavonoid PA sakuranetin shares the roles and collaborates with diterpenoid PAs to restrict blast fungus. The different characteristics of individual rice PA may guarantee the diversity of the resistance to blast fungus.
